# Objectively Measured Smartphone Use and Nonsuicidal Self-Injury Among College Students: Cross-Sectional Study

**DOI:** 10.2196/71264

**Published:** 2025-07-30

**Authors:** Wenhua Wang, Mingyang Wu, Xiaoxiao Yuan, Xue Wang, Le Ma, Lu Li, Lei Zhang

**Affiliations:** 1 Department of Gastroenterology, Second Affiliated Hospital of Xi'an Jiaotong University, 157 West Five Road, Xi'an, 710004, China, +86-158-0918-5895; 2 Shaanxi Provincial Health Industry Association Service Center, Xi'an, China; 3 Xiangya School of Public Health, Central South University, Changsha, China; 4 School of Public Health, Xi'an Jiaotong University, Xi'an, China; 5 Shaanxi Medical Assocaition, Xi'an, China

**Keywords:** smartphone screen time, number of smartphone unlocks, nonsuicidal self-injury, objective, college students

## Abstract

**Background:**

The impact of smartphone use on mental health is being rigorously debated. Some questionnaire-based research suggests that smartphone use correlates with nonsuicidal self-injury (NSSI). Self-reported data seem unlikely to capture actual smartphone use precisely, requiring objective measures to advance this field.

**Objective:**

The aim of the study is to examine whether objectively measured smartphone use was associated with NSSI among college students.

**Methods:**

This multicenter cross-sectional study was conducted from 2022 to 2024, enrolling college students from 559 classes across 6 universities in China. NSSI was measured by the Ottawa Self-Injury Inventory including 10 items of NSSI without suicidal intent within the past month. Participants answering “ever” were classified as having NSSI. Objectively measured smartphone screen time and number of smartphone unlocks were obtained from screenshots of smartphone use records. The association between objectively measured smartphone use and NSSI was analyzed using binary logistic regression models and restricted cubic spline regression.

**Results:**

Of 16,668 included participants, 627 (3.8%) reported NSSI. Mean (SD) smartphone screen time and number of smartphone unlocks were 48.8 (28.8) hours per week and 271.6 (291.0) times per week. The models adjusted for different factors showed a significant association between smartphone use and NSSI. Compared to participants with 0-21 hours per week of smartphone screen time, those with ≥63 hours per week of smartphone screen time had higher odds of NSSI (odds ratio [OR] 1.63, 95% CI 1.32‐2.01). Likewise, compared to participants with 0-50 times per week of smartphone unlocks, those with ≥400 times per week of smartphone unlocks had higher odds of NSSI (OR 1.53, 95% CI 1.25‐1.88). No significant NSSI risk increase was observed for participants with 21-42 and 42-63 hours per week of smartphone screen time nor for those with 50-150 and 150-400 times per week of smartphone unlocks. Moreover, restricted cubic spline analyses showed that the increasing risk of NSSI was associated with elevated smartphone screen time and number of smartphone unlocks.

**Conclusions:**

These findings emphasize that ≥63 hours per week of smartphone screen time and ≥400 times per week of smartphone unlocks are risk factors for NSSI among college students, and interventions targeting NSSI should consider the apparent association with smartphone use.

## Introduction

The role of smartphone use in mental health has been rigorously debated by the public and academia [1,2]. Some literature suggests that excessive smartphone use is related to poor mental health conditions [3-5], such as depression, anxiety, and sleep disorders. However, other evidence implies that adverse daily life outcomes of smartphone use may be overestimated [1]. Much of the discourse regarding smartphone use and mental health has focused on smartphone addiction and dependence, which typically reflect the subjective experience of smartphone use. Smartphones are now a ubiquitous part of everyday life. The prevalence of smartphone ownership and excessive smartphone use has increased exponentially in recent years among both youths and adults. The widespread use of smartphones in everyday life points to the need for rigorous and objective methods that can adequately capture the true association between smartphone use behaviors and mental health. Several investigations have explored associations between smartphone use behaviors—including smartphone screen time of use, number of smartphone unlocks, and text message volume—and mental health outcomes [5-8]. Nevertheless, most research relied on participants’ self-reported smartphone use [6-8]. This is an important limitation because prior studies showed that self-reported smartphone use has significant recall bias and misclassification probability [9-11], and such self-reporting does not correlate well with objectively measured smartphone use. Moreover, it should be particularly noted that using self-reported methods to evaluate the unconscious behavior of smartphone use may be especially inappropriate for people with psychological problems, as they are likely to either underestimate or overestimate their actual smartphone use behaviors [9].

Nonsuicidal self-injury (NSSI) refers to the intentional act of harming one’s own body without the aim of ending life [12]. NSSI is a complex phenomenon with a wide variety of underlying causes and contributing factors. Repeated engaging in NSSI can desensitize individuals to sensations of pain, harm, fear, and mortality, thus increasing the likelihood of suicide attempts [13]. Notably, unlike suicide prevention, NSSI has been neglected by governments internationally. The association between smartphone use and NSSI may be especially relevant for college students, who tend to exhibit more severe and widespread excessive smartphone use. Indeed, during the crucial phase of transitioning to adulthood, college students are particularly prone to external stressors, including pressures from peers, academic responsibilities, employment pressure, and social anxiety [14]. They exhibit a high risk of NSSI, albeit lower than middle and high school students. The social learning hypothesis of Nock’s integrated model of NSSI also believes that observing others’ NSSI behavior can prompt individuals to translate NSSI ideation into actual action [15]. With the widespread adoption of smartphones among college students, it has also become the primary means for them to obtain information about NSSI, learn NSSI methods, and connect with peers who are involved in NSSI. Additionally, the vivid and repetitive descriptions of pleasure and harming ways on smartphones may lead to an underestimation of the seriousness and consequences of NSSI [16]. Some literature suggests that excessive smartphone use can supplant sleep and physical activity [17] as well as social interaction [18,19], all of which are known to be beneficial to mental health. Moreover, smartphone use might interfere with self-regulation strategies [20], which may be correlated with NSSI.

To our knowledge, little research has examined the association between excessive smartphone use and NSSI. The extant studies [21-25], primarily based on self-reported surveys, have connected smartphone addiction or dependence to NSSI. Only 2 studies specifically investigated the relationship between self-reported time spent on smartphones and NSSI in middle school student populations [21,22]. To date, no research has examined the role of smartphone use behaviors in NSSI among college students or used methods that can measure objective smartphone use behaviors. Emerging conceptual frameworks posit smartphone use as a multidimensional construct with differential mental health impacts. Smartphone screen time and number of smartphone unlocks represent distinct aspects of smartphone use behavior [26,27]. Specifically, smartphone screen time quantifies the cumulative duration of active or passive content consumption, whereas the number of smartphone unlocks reflects habitual checking behaviors. Compared with smartphone screen time, number of smartphone unlocks involves compulsive, notification-driven interactions and is more strongly associated with anxiety, addiction, and problematic use patterns. What is more, prior findings on the associations of these 2 smartphone metrics and mental health have been inconsistent [28-30]. A study using the objective method showed that objective smartphone time and number of smartphone unlocks might reflect differences in smartphone use patterns [28]. Specifically, it revealed that depression and anxiety severity were correlated with the number of smartphone unlocks but not related to smartphone screen time. While a recent study using smartphone sensing technology found that longer smartphone screen time predicted higher internalizing and externalizing symptoms in youths, more smartphone unlocks were associated with lower internalizing symptoms [29]. Notably, another investigation reported a positive association between the number of screen unlocks and loneliness [30]. However, the sample sizes of these studies were extremely limited, with a maximum of only a few hundred participants. Adding to the limited relevant studies at present, further research is required.

This study assessed objective smartphone screen time and number of smartphone unlocks by collecting screenshots of participants’ smartphone use records, with the aim of examining whether objectively measured smartphone use was associated with NSSI in college students. Furthermore, we performed subgroup analyses by sex and grade to better understand the relationship between smartphone use and NSSI within this crucial demographic.

## Methods

### Ethical Considerations

The Second Affiliated Hospital of Xi’an Jiaotong University granted ethics approval for this research (approval ID: 2022‐248). All participants gave their electronic informed consent prior to participating in the study. The study adhered to the STROBE (Strengthening the Reporting of Observational Studies in Epidemiology) reporting guideline. Data were analyzed from December 2023 to October 2024. The identifiable features of participants in the paper or Multimedia Appendix 1 are not visible.

### Recruitment

In this cross-sectional study, we collected data from a representative group of college students in Shaanxi province, located in Northwest China. The study design and investigation process have been previously described [31]. First, 6 universities were randomly selected from a pool of 57 universities of Shaanxi province, comprising 4 public universities of 34 and 2 private universities of 23. Afterward, for each of the universities under investigation, we randomly selected 2 to 4 classes from each grade within every department or college. All students enrolled in these chosen classes were encouraged to take part in the survey. The consistency of the proportion of students participating in the survey across universities was maintained by adjusting the number of selected classes. Prior to the investigation, we organized a 2-phase training to explain the objectives and processes to the students, including training of 2 officers from each chosen class, who then trained all other students in their respective classes.

In total, 20,165 undergraduates from 559 classes at 6 universities were invited to join in the study. We included all 16,668 students who provided clear screenshots of their smartphone use records.

### Measures

#### Objectively Measured Smartphone Use

In this study, the objective smartphone use included smartphone screen time and number of smartphone unlocks. We gathered the objective smartphone use data by collecting screenshots of participants’ smartphone use records. Two weeks before the formal investigation, we instructed the participants to activate the function for smartphone records to ensure the availability of objective data. Then, we provided step-by-step instructions for finding screenshots of smartphones with different brands and types and required them to submit the screenshots covering (1) smartphone screen time during the past week and (2) number of smartphone unlocks during the past week. According to the present distribution of data and previous studies on smartphone screen time [32-34], we classified participants into 4 groups (0-21, 21-42, 42-63, and ≥63 hours per week). To ensure that the analysis aligns with our dataset distribution and offers practical interpretability, we partitioned smartphone unlocks into quartiles using integer thresholds. Consequently, we classified participants into 4 groups for number of smartphone unlocks (0-50, 50-150, 150-400, and ≥400 times per week).

#### Nonsuicidal Self-Injury

The Ottawa Self-Injury Inventory, a commonly used instrument, was used to assess NSSI [35]. It comprises 10 NSSI behaviors, including hitting, head banging, stabbing or cutting, pinching, scratching, biting, burning, drowning, excessive or indiscriminate medication use, and swallowing or drinking inedible items. Participants who responded “ever” during the past month were classified as having NSSI. The Ottawa Self-Injury Inventory has demonstrated both validity and reliability within the Chinese population [36]. In this study, the Cronbach α coefficient was 0.94.

#### Covariates

We developed a structured questionnaire to collect several confounding factors that have been recognized as relevant to both smartphone use and NSSI [37,38], including sociodemographic information, health-related lifestyles, and negative life events. Sociodemographic information incorporated sex, grade, race, registered permanent residence, siblings, and parental educational attainment.

As described in a previous study [39], health-related lifestyles included current smoking, current drinking, physical activity, and rational diet. Participants who smoked at least 1 cigarette in the last 30 days were classified as current smokers. Similarly, those who consumed at least 1 glass of wine during the past 30 days were categorized as current drinking. We used the International Physical Activity Questionnaire Short Form to assess physical activity, categorizing activity levels into low, moderate, and high according to established metabolic equivalent calculations. Irrational diet was defined as participants consuming red meat every day or vegetables or fruits less than daily. The negative life events included family disasters, hospitalization experiences, failed examinations, and failed relationships within the last year.

### Statistical Analyses

Frequencies and proportions for categorical variables, as well as mean and SD for continuous variables, were used to depict the characteristics of participants. We calculated odds ratios (ORs) and 95% CIs for NSSI using binary logistic regression models, with adjustment for sex, grade, race, registered permanent residence, siblings, parental educational attainment, current smoking, current drinking, physical activity, rational diet, family disasters, hospitalization experience, failed examinations, and failed relationships. Restricted cubic spline regression was used to estimate the dose-response relationship between objectively measured smartphone use and NSSI, with knots optimized by minimizing both the Akaike information criterion and Bayesian information criterion. Furthermore, we tested for separate interactions between objectively measured smartphone use and (1) sex and (2) grade to determine whether these variables modify the connection between objectively measured smartphone use and NSSI. In addition, chi-square and Spearman correlation tests were used to estimate the relationship between smartphone screen time and number of smartphone unlocks.

Sensitivity analyses were conducted to check the robustness of our estimations. First, to reduce the possible effects of the unbalanced sex ratio of participants, we performed a weighted logistic regression. Second, to ensure that the use of different measurement time frames of NSSI (eg, NSSI within the past month or 12 months) did not affect the results, we analyzed the association of objectively measured smartphone use with NSSI during the past 12 months. Third, to mitigate the class clustering effects, we performed multilevel modeling with classes as random effects.

In all tests, a 2-sided *P*<.05 was used as the significance threshold. R software (version 4.0.2; R Foundation for Statistical Computing) was used for all data analyses.

## Results

Table 1 presents the characteristics of participants involved in the study. Of the 16,668 participants, 5881 (35.3%) were male, and 10,787 (64.7%) were female. A total of 16,169 (97%) were Han ethnicity, 8993 (54%) from rural, and 4940 (29.6%) from single-child families (Table 1). Notably, 627 (3.8%) reported committing NSSI during the past month. Overall, the mean smartphone screen time was 48.8 (SD 28.8) hours per week, and the mean number of smartphone unlocks was 271.6 (SD 291.0) times per week.

**Table 1.** Characteristics of participants.

Characteristics	Participants	NSSI^a^		*P* value
Never	Ever
Sex, n (%)				.30
Male	5881 (35.3)	5672 (96.4)	209 (3.6)	
Female	10,787 (64.7)	10,369 (96.1)	418 (3.9)	
Grade, n (%)				.01
First	4892 (29.3)	4740 (96.9)	152 (3.1)	
Second	3907 (23.5)	3736 (95.6)	171 (4.4)	
Third	3913 (23.5)	3757 (96)	156 (4)	
Fourth+	3956 (23.7)	3808 (96.3)	148 (3.7)	
Race, n (%)				.44
Han	16,169 (97)	15,564 (96.3)	605 (3.7)	
Others	499 (3)	477 (95.6)	22 (4.4)	
Registered permanent residence, n (%)				.003
Rural	8993 (54)	8691 (96.6)	302 (3.4)	
Urban	7675 (46)	7350 (95.8)	325 (4.2)	
Siblings, n (%)				.13
No	4940 (29.6)	4737 (95.9)	203 (4.1)	
Yes	11,728 (70.4)	11,304 (96.4)	424 (3.6)	
Maternal educational attainment, n (%)				.26
Middle school or under	10,726 (64.4)	10,339 (96.4)	387 (3.6)	
High school	3401 (20.4)	3270 (96.1)	131 (3.9)	
College or above	2541 (15.2)	2432 (95.7)	109 (4.3)	
Parental educational attainment, n (%)				.45
Middle school or under	9123 (54.7)	8794 (96.4)	329 (3.6)	
High school	3697 (22.2)	3547 (95.9)	150 (4.1)	
College or above	3848 (23.1)	3700 (96.2)	148 (3.8)	
Current smoking, n (%)				<.001
No	14,700 (88.2)	14,195 (96.6)	505 (3.4)	
Yes	1968 (11.8)	1846 (93.8)	122 (6.2)	
Current drinking, n (%)				<.001
No	13,485 (80.9)	13,097 (97.1)	388 (2.9)	
Yes	3183 (19.1)	2944 (92.5)	239 (7.5)	
Rational diet, n (%)				.48
Yes	1294 (7.8)	1250 (96.6)	44 (3.4)	
No	15,374 (92.2)	14,791 (96.2)	583 (3.8)	
Physical exercise, n (%)				.30
Moderate or high	4446 (26.7)	4290 (96.5)	156 (3.5)	
Low	12,222 (73.3)	11,751 (96.1)	471 (3.9)	
Family disasters, n (%)				<.001
No	15,041 (90.2)	14,513 (96.5)	528 (3.5)	
Yes	1627 (9.8)	1528 (93.9)	99 (6.1)	
Hospitalization experience, n (%)				<.001
No	15,266 (91.6)	14,762 (96.7)	504 (3.3)	
Yes	1402 (8.4)	1279 (91.2)	123 (8.8)	
Failed examinations, n (%)				<.001
No	8549 (51.3)	8322 (97.3)	227 (2.7)	
Yes	8119 (48.7)	7719 (95.1)	400 (4.9)	
Failed relationships, n (%)				<.001
No	13,642 (81.8)	13,229 (97)	413 (3)	
Yes	3026 (18.2)	2812 (92.9)	214 (7.1)	
Smartphone screen time (hours per week), n (%)				<.001
0-21	3707 (22.3)	3586 (96.7)	121 (3.3)	
21-42	2818 (16.9)	2737 (97.1)	81 (2.9)	
42-63	4322 (25.9)	4176 (96.6)	146 (3.4)	
≥63	5821 (34.9)	5542 (95.2)	279 (4.8)	
Smartphone screen time (hours per week), mean (SD)	48.8 (28.8)	48.6 (28.8)	54.2 (29.6)	<.001
Number of smartphone unlocks (times per week), n (%)				<.001
0-50	3525 (21.1)	3420 (97)	105 (3)	
50-150	4766 (28.6)	4621 (97)	145 (3)	
150-400	4091 (24.6)	3929 (96)	162 (4)	
≥400	4286 (25.7)	4071 (95)	215 (5)	
Number of smartphone unlocks (times per week), mean (SD)	271.6 (291.0)	269.0 (288.8)	339.2 (336.3)	<.001

a NSSI: nonsuicidal self-injury.

We observed positive associations between smartphone screen time and number of smartphone unlocks with NSSI among college students (Table 2). In the model adjusted for sociodemographic factors (Table 2, model 1), participants with ≥63 hours per week of smartphone screen time had significantly higher odds of NSSI (OR 1.63, 95% CI 1.32‐2.01) as compared to those with 0‐21 hours per week of smartphone screen time. Likewise, compared to participants with 0-50 times per week of smartphone unlocks, those with ≥400 times per week of smartphone unlocks had higher odds of NSSI (OR 1.53, 95% CI 1.25‐1.88). A 21 hours per week increase in smartphone screen time (OR 1.18, 95% CI 1.11‐1.25) and a 50 times per week increase in the number of smartphone unlocks (OR 1.04, 95% CI 1.03‐1.05) were significantly associated with greater likelihoods of NSSI. In the model adjusted for sociodemographic factors and health-related lifestyles (Table 2, model 2), the associations between objectively measured smartphone use and NSSI were slightly attenuated. In the model fully adjusted for sociodemographic factors, health-related lifestyles, and negative life events (Table 2, model 3), compared with participants with 0-21 hours per week of smartphone screen time, those with ≥63 hours per week of smartphone screen time had significantly higher odds of NSSI (OR 1.54, 95% CI 1.25‐1.90), those with 21-42 hours per week (OR 0.96, 95% CI 0.73‐1.26) and 42‐63 hours per week (OR 1.12, 95% CI 0.88‐1.41) of smartphone screen time exhibited no significant increase in NSSI odds. A 21 hours per week increase in smartphone screen time was significantly associated with a greater likelihood of NSSI (OR 1.15, 95% CI 1.08‐1.23). Regarding the number of smartphone unlocks, participants with ≥400 times per week of number of smartphone unlocks had significantly higher NSSI odds (OR 1.43, 95% CI 1.16‐1.77) than those with 0-50 times per week, whereas for those with 50‐150 times per week (OR 0.91, 95% CI 0.73‐1.14) and 150-400 times per week (OR 1.15, 95% CI 0.92‐1.43), number of smartphone unlocks showed no significant increase in NSSI risk. A 50 times per week increase in the number of smartphone unlocks was significantly linked to a higher NSSI likelihood (OR 1.03, 95% CI 1.02‐1.04).

**Table 2.** Associations between objectively measured smartphone use and NSSI^a^.

Objectively measured smartphone use	OR^b^ (95% CI)^c^		
	Model 1^d^	Model 2^e^	Model 3^f^
Smartphone screen time (hours per week), per 21 hours per week	1.18 (1.11‐1.25)	1.16 (1.09‐1.24)	1.15 (1.08‐1.23)
0-21	1 (reference)	1 (reference)	1 (reference)
21-42	0.91 (0.69‐1.19)	0.94 (0.71‐1.23)	0.96 (0.73‐1.26)
42-63	1.13 (0.90‐1.42)	1.12 (0.89‐1.42)	1.12 (0.88‐1.41)
≥63	1.63 (1.32‐2.01)	1.59 (1.29‐1.96)	1.54 (1.25‐1.90)
Number of smartphone unlocks (times per week), per 50 times per week	1.04 (1.03‐1.05)	1.03 (1.02‐1.05)	1.03 (1.02‐1.04)
0-50	1 (reference)	1 (reference)	1 (reference)
50-150	0.92 (0.74‐1.15)	0.89 (0.71‐1.12)	0.91 (0.73‐1.14)
150-400	1.16 (0.93‐1.45)	1.12 (0.90‐1.40)	1.15 (0.92‐1.43)
≥400	1.53 (1.25‐1.88)	1.44 (1.17‐1.77)	1.43 (1.16‐1.77)

a NSSI: nonsuicidal self-injury

b OR: odds ratio.

c *P* for trend<.001.

d Model 1: adjusted for sex, grade, race, registered permanent residence, siblings, and parental educational attainment.

e Model 2: adjusted for sex, grade, race, registered permanent residence, siblings, parental educational attainment, current smoking, current drinking, physical activity, and rational diet.

f Model 3: adjusted for sex, grade, race, registered permanent residence, siblings, parental educational attainment, current smoking, current drinking, physical activity, rational diet, family disasters, hospitalization experience, failed exams, and failed relationships.

**Figure 1.**
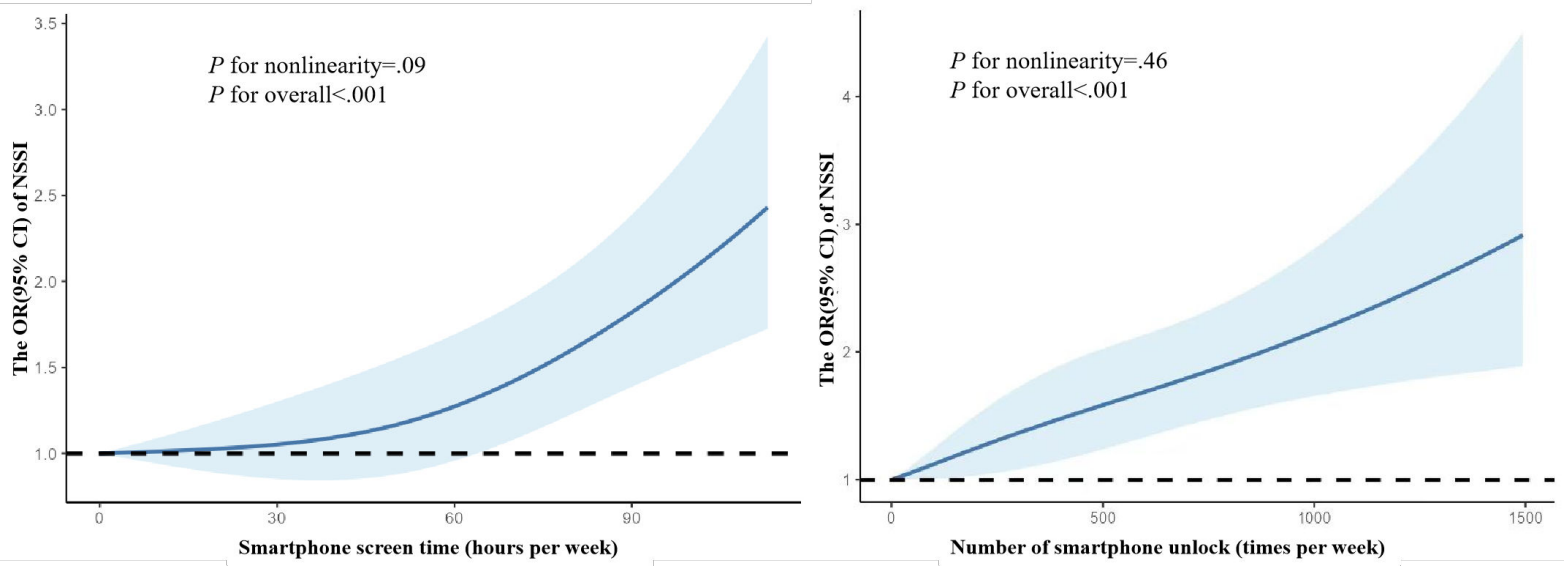
Restricted cubic spline regression analysis of objectively measured smartphone use with NSSI risk. Adjusted for sex, grade, race, registered permanent residence, siblings, parental educational attainment, current smoking, current drinking, physical activity, rational diet, family disasters, hospitalization experience, failed examinations, and failed relationships. NSSI: nonsuicidal self-injury; OR: odds ratio.

In all models, increasing smartphone screen time and number of smartphone unlocks were significantly associated with higher risks of NSSI, with all trend tests significant (all *P* for trend<.05; Table 2). We used a restricted cubic spline to flexibly model and visualize the relationships. The results suggested a positive association between NSSI risk and increasing smartphone screen time. The risk of NSSI was increased relatively slowly until approximately 45 hours per week of smartphone screen time, after which it showed an accelerated upward trend (*P* for nonlinearity*=*.09). By contrast, NSSI risk exhibited a monotonically increasing trend with a number of smartphone unlocks (*P* for nonlinearity=.46; Figure 1).

The results of sex-stratified and grade-stratified analyses are presented in Table 3. Objectively measured smartphone use was related to NSSI both in male and female participants. Male participants (OR 1.96, 95% CI 1.35‐2.84) with ≥63 hours per week of smartphone screen time had a higher risk of NSSI compared to female participants (OR 1.36, 95% CI 1.05‐1.76). Regarding the association between the number of smartphone unlocks and NSSI, it was significant among female participants but not so significant among male participants. While no significant sex-specific difference was observed in the associations (all *P* for interaction*>*.05). Grade-stratified analyses showed that associations between smartphone screen time and NSSI were significant among freshmen (OR 2.14, 95% CI 1.33‐3.43 for ≥63 hours per week of smartphone screen time vs 0-21 hours per week) and sophomores (OR 1.83, 95% CI 1.24‐2.71 for ≥400 times per week of smartphone unlocks vs 0-50 times per week) but not evident among juniors, seniors, and higher grade students. However, the grade interaction effect was also not significant (all *P* for interaction>.05). Similar patterns were observed for the number of smartphone unlocks (Table 3).

**Table 3.** Sex-specific and grade-specific associations of objectively measured smartphone use with NSSI^a^^b^.

Objectively measured smartphone use	Male		Female		Grade first		Grade second		Grade third		Grade fourth+	
Values, n (%)	OR^c^ (95% CI)	Values, n (%)	OR (95% CI)	Values, n (%)	OR (95% CI)	Values, n (%)	OR (95% CI)	Values, n (%)	OR (95% CI)	Values, n (%)	OR (95% CI)
Smartphone screen time (hours per week)^d^												
0-21	1495 (25.4)	1 (reference)	2212 (20.5)	1 (reference)	1024 (20.9)	1 (reference)	915 (23.4)	1 (reference)	823 (21)	1 (reference)	945 (23.9)	1 (reference)
21-42	1073 (18.3)	1.17 (0.73‐1.86)	1745 (16.2)	0.87 (0.62‐1.23)	702 (14.4)	1.41 (0.77‐2.58)	573 (14.7)	0.77 (0.42‐1.41)	596 (15.3)	1.15 (0.67‐1.98)	947 (23.9)	0.73 (0.44‐1.20)
42-63	1488 (25.3)	1.34 (0.89‐2.02)	2834 (26.3)	1.01 (0.75‐1.34)	1141 (23.3)	1.19 (0.68‐2.09)	988 (25.3)	1.14 (0.72‐1.81)	1084 (27.7)	1.07 (0.66‐1.74)	1109 (28)	1.12 (0.73‐1.71)
≥63	1825 (31)	1.96 (1.35‐2.84)	3996 (37)	1.36 (1.05‐1.76)	2025 (41.4)	2.14 (1.33‐3.43)	1431 (36.6)	1.83 (1.24‐2.71)	1410 (36)	1.40 (0.91‐2.17)	955 (24.2)	1.10 (0.71‐1.71)
Number of smartphone unlocks (times per week)^e^												
0-50	1182 (20.1)	1 (reference)	2343 (21.7)	1 (reference)	1101 (22.5)	1 (reference)	826 (21.2)	1 (reference)	785 (20.1)	1 (reference)	813 (20.6)	1 (reference)
50-150	1755 (29.8)	0.82 (0.55‐1.22)	3011 (27.9)	0.96 (0.73‐1.26)	1424 (29.1)	1.27 (0.80‐2.02)	1223 (31.3)	1.33 (0.87‐2.02)	1055 (26.9)	0.57 (0.35‐0.92)	1064 (26.9)	0.68 (0.42‐1.10)
150-400	1387 (23.6)	1.32 (0.89‐1.94)	2704 (25.1)	1.07 (0.81‐1.40)	1089 (22.3)	1.32 (0.81‐2.16)	943 (24.1)	1.23 (0.78‐1.94)	1009 (25.8)	1.09 (0.71‐1.66)	1050 (26.5)	1.05 (0.68‐1.61)
≥400	1557 (26.5)	1.24 (0.85‐1.80)	2729 (25.3)	1.54 (1.19‐1.99)	1278 (26.1)	1.73 (1.11‐2.71)	915 (23.4)	2.01 (1.33‐3.05)	1064 (27.2)	1.08 (0.72‐1.63)	1029 (26)	1.23 (0.81‐1.88)

a NSSI: nonsuicidal self-injury.

b This analysis was adjusted for sex, grade, race, registered permanent residence, siblings, parental educational attainment, current smoking, current drinking, physical activity, rational diet, family disasters, hospitalization experience, failed examinations, and failed relationships.

c OR: odds ratio.

d *P* value for trend (male): .002; *P* value for trend (female): .006; *P* value for trend (grade first): .003; *P* value for trend (grade second): <.001; *P* value for trend (grade third): .39; *P* value for trend (grade fourth+): .32. *P* value for interaction (male and female): .40; *P* value for interaction (grade first): .16.

e *P* value for trend (male): .08; *P* value for trend (female): <.001; *P* value for trend (grade first): <.001; *P* value for trend (grade second): .008; *P* value for trend (grade third): .04; *P* value for trend (grade fourth+): .11. *P* value for interaction (male and female): .48; *P* value for interaction (grade first): .71.

In the sex-weighted logistic regression model, objectively measured smartphone use remained significantly associated with NSSI (Table S1 in Multimedia Appendix 1). The results of sensitivity analyses were comparable when NSSI was defined as having occurred within the last 12 months (Table S2 in Multimedia Appendix 1). Multilevel modeling with classes as random effects showed that the significant association between objectively measured smartphone use and NSSI was also not changed materially (Table S3 in Multimedia Appendix 1). What is more, there was a moderate correlation between smartphone screen time and number of smartphone unlocks (*χ*²_9_=3209.2; *P*<.001*; r*=0.347; *P*<.001; Table S4 and S5 in Multimedia Appendix 1).

## Discussion

### Principal Findings

To our knowledge, this is the largest study to date to measure actual smartphone use using an objective method and the first study to comprehensively analyze the association between objectively measured smartphone use and NSSI among college students. College students with longer smartphone screen time and more smartphone unlocks showed a higher risk of NSSI.

### Comparison to Prior Work

In recent years, smartphones have penetrated various aspects of daily lives. Self-reported surveys commonly used to measure smartphone use have notoriously unreliable estimates of use time and unlock frequency. This study makes an important contribution to the extant literature because of the extreme deficiency in objectively measured smartphone use data, particularly for Chinese populations, where smartphone ownership and addiction rates are at the forefront globally [40]. This study found that the average smartphone screen time and number of smartphone unlocks, measured by screenshots of participants’ smartphone use records, were significantly higher than previous self-reported smartphone use among college students [41,42]. They were also much higher than the results reported in some previous studies with objective measurement. For instance, a study from the United States measured smartphone use of 99 college students using iOS’ ScreenTime feature and found that the average smartphone screen time is 5.48 (SD 2.18) hours per day [10]. Rozgonjuk et al [28] tracked the objective smartphone screen time and unlocked number of 101 college students by installing the Moment app on their Apple phones, reporting that an average objective smartphone use time was 4.05 (SD 1.72) hours per day. The differences in the above research findings further indicate the necessity of using objective measurements of smartphone use behavior in future studies.

To our knowledge, few studies have evaluated the association between smartphone use and NSSI [21-23]. For example, a study found that self-reported high-intensity smartphone use predicted NSSI in middle and high school students [21]. Another Japanese study also reported that smartphone use after lights out increases the NSSI of adolescents [22]. However, neither of the 2 studies was based on objective data. Bye et al [23] proposed a protocol for a 6-month cohort study of 600 participants, which extracted smartphone metadata by installing apps on the participants’ smartphones to analyze the correlation between smartphone use and self-harm. To date, the results remain unpublished. Our investigation provides an important contribution by exploring the relationship between objectively measured smartphone use and NSSI. Moreover, the research showed that there was no significant sex and grade disparity in the association between smartphone use and NSSI, which was unexpected [43] but mirrors several similar studies [25,44]. The challenges of excessive smartphone use and NSSI are continuously intensifying. Our findings confirm this positive association, suggesting that addressing excessive smartphone use is important in college students’ NSSI initiatives.

### Possible Explanations of the Associations

Several factors may contribute to the association. First, the results in this study align with the integrated motivation-volition model of NSSI [15]. The model proposes that an individual’s decision to act on NSSI ideation depends on having the capability, knowing others who engage in NSSI, and having access to means. People who spend more time on smartphones are more likely to come across peers engaged in NSSI and relevant information, increasing the propensity to imitate. Indeed, a study reported that 43% learn NSSI from others, and 21% from media [45]. Second, both NSSI and excessive smartphone use serve as emotion regulation strategies [20,46], which may be another reason for the association. The compensatory internet use theory suggests that individuals under stress or experiencing negative emotions frequently resort to using technology, such as smartphone use, to relieve emotional discomfort [47]. According to the four-function model of NSSI [48], emotion regulation was one of the most important functions of NSSI. The practice of NSSI can trigger acute physiological experiences, and internal emotional issues can be alleviated by being redirected to the physiological experiences of NSSI. Third, sleep is another possible reason for the association. Prolonged smartphone screen time and more smartphone unlocks can decrease sleep quality and shorten sleep duration, both of which are risk factors for NSSI [49,50]. Finally, social support plays an important role. Prolonged smartphone use time and more smartphone unlocks will take up large time and attention, reducing real-world interaction and objective social support [51]. In addition, negative views of college students’ excessive smartphone use also greatly impair subjective support [18]. While the protective effect of high social support on NSSI has been widely recognized [52]. Previous research found that interpersonal problems act as a mediator in the connection between excessive smartphone use and NSSI risk [21].

Notably, although statistical analysis suggested a linear dose-response relationship for both smartphone screen time and number of smartphone unlocks with NSSI, the response curve for smartphone screen time indicated a potential nonlinear association with NSSI (*P* for nonlinearity=.09). Given the limited prior research, the underlying reasons for the discrepancy in the *P* for nonlinearity remain unclear. We posit that this divergence is likely rooted in the distinct behavioral meanings of these 2 metrics and the nuanced app-specific use patterns. First, smartphone screen time quantifies cumulative user engagement—encompassing both active and passive content consumption—and serves as a proxy for use intensity. Some prior literature suggests that excessive smartphone screen time is related to depression and anxiety [4,53], while 2 other studies showed that smartphone nonusers are more likely to experience depression and anxiety [54,55]. The marginal *P* for nonlinearity between smartphone screen time and NSSI in this study appears to align with this nonlinear hypothesis. In contrast, the number of smartphone unlocks reflects compulsive device-checking behavior, often driven by notification dependency or nomophobia (the fear of disconnection), which is a core component of smartphone addiction [56]. Such behavior is tightly linked to reward-seeking loops (eg, dopamine-driven responses to notifications) [57]. Unlike smartphone screen time—which may involve passive activities such as video-watching with minimal interaction—unlocks represent inherently active, goal-directed behaviors such as communicating via social media. The *P* for nonlinearity of .46 in this study suggests that each additional smartphone unlock reinforces compulsive tendencies, cumulatively increasing NSSI vulnerability in a dose-dependent manner. Additionally, smartphone screen time encompasses diverse app uses (eg, social media, gaming, and productivity tools), each carrying distinct mental health implications. Passive consumption (eg, endless browsing of the internet) may induce rumination, whereas purposeful use (eg, communicating via social media) could exert protective effects [29,58]. This heterogeneity in smartphone screen time composition may dilute potential nonlinear signals, leading to marginally significant statistical findings. By contrast, the number of smartphone unlocks, which is more directly associated with addictive mechanisms, is likely less affected by the behavioral diversity inherent in smartphone screen time metrics. Future research should therefore dissect the distinct pathways through which these behaviors influence NSSI risk, alongside the nuanced impacts of specific app use patterns.

### Limitations, Strengths, and Implications

The following limitations should be noted: first, although the models included a wide range of demographic and lifestyle factors, some confounding variables may have remained unaccounted for, potentially influencing the study findings. Second, a retrospective questionnaire was used to assess NSSI behavior, which may result in recall bias. Third, due to the cross-sectional study design, it is difficult to infer whether excessive smartphone use leads to NSSI or vice versa, which should be addressed in future studies. Fourth, although this study measured smartphone use time and frequency objectively, it did not specify the functional types of smartphone use, which may have implications for mental health. Finally, although the multilevel model treating class membership as a random effect yielded substantially unchanged results, the study did not account for clustering effects stemming from shared living or social environments (eg, dormitories and clubs). Future research could address these clustering effects by implementing simple random sampling methods, which would help minimize potential biases arising from nonindependent observations.

### Conclusions

This study suggests a significant association between excessive smartphone use and NSSI. These findings have important implications for public health and school-based interventions, highlighting the importance of evaluating NSSI risk among adolescents with excessive smartphone use. In line with extant clinical guidelines, taking smartphone use into account when working with youths engaged in NSSI is important and may help to facilitate treatment.

## Supplementary material

10.2196/71264 Multimedia Appendix 1

## Abbreviations

NSSI: nonsuicidal self-injury
OR: odds ratio
STROBE: Strengthening the Reporting of Observational Studies in Epidemiology
