# Supplementary material for: Objectively Measured Smartphone Use and Nonsuicidal Self-Injury Among College Students: Cross-Sectional Study
Source: JMIR Ment Health. 2025 Jul 30;12:e71264. doi: 10.2196/71264 (PMC12310150; doi:10.2196/71264)
Supplement: Multimedia Appendix 1 [file mental-v12-e71264-s001.docx]

**Table S1.** Associations between objectively measured smartphone use and NSSI in gender weighted model.

| Objectively measured smartphone use | *OR (95%CI)* | | |
| --- | --- | --- | --- |
|  | Model 1 | Model 2 | Model 3 |
| Smartphone screen time (hours/week) |  |  |  |
| 0~21 | 1[Reference] | 1[Reference] | 1[Reference] |
| 21~42 | 0.87(0.70-1.08) | 0.90(0.73-1.12) | 0.92(0.75-1.14) |
| 42~63 | 1.09(0.91-1.30) | 1.07(0.90-1.29) | 1.07(0.89-1.29) |
| ≥63 | 1.56(1.33-1.83) | 1.52(1.29-1.79) | 1.47(1.25-1.73) |
| *P_trend_* | <.001 | <.001 | <.001 |
| Number of smartphone unlocks (times/week) |  |  |  |
| 0~50 | 1[Reference] | 1[Reference] | 1[Reference] |
| 50~150 | 0.94(0.79-1.11) | 0.91(0.76-1.08) | 0.93(0.78-1.11) |
| 150~400 | 1.13(0.95-1.34) | 1.09(0.92-1.30) | 1.11(0.94-1.32) |
| ≥400 | 1.57(1.34-1.83) | 1.48(1.26-1.74) | 1.48(1.26-1.74) |
| *P_trend_* | <.001 | <.001 | <.001 |

**Note:** NSSI, non-suicidal self-injury; OR, odds ratio; CI, confidence interval.

Model 1, adjusted for gender, grade, race, registered permanent residence, siblings, and parental educational attainment.

Model 2, adjusted for gender, grade, race, registered permanent residence, siblings, parental educational attainment, current smoking, current drinking, physical activity, and rational diet.

Model 3, adjusted for gender, grade, race, registered permanent residence, siblings, parental educational attainment, current smoking, current drinking, physical activity, rational diet, family disasters, hospitalization experience, failed exams, and failed relationships.

**Table S2.** Associations between objectively measured smartphone use and NSSI during the past 12 months.

| Objectively measured smartphone use | *OR (95%CI)* | | |
| --- | --- | --- | --- |
|  | Model 1 | Model 2 | Model 3 |
| Smartphone screen time (hours/week) |  |  |  |
| 0~21 | 1[Reference] | 1[Reference] | 1[Reference] |
| 21~42 | 1.02(0.83-1.26) | 1.05(0.85 1.30) | 1.08(0.87 1.33) |
| 42~63 | 1.20(1.00-1.44) | 1.20(0.99 1.44) | 120(1.99 1.44) |
| ≥63 | 1.68(1.43-1.98) | 1.65(1.40 1.95) | 1.61(1.36 1.91) |
| *P_trend_* | <.001 | <.001 | <.001 |
| Number of smartphone unlocks (times/week) |  |  |  |
| 0~50 | 1[Reference] | 1[Reference] | 1[Reference] |
| 50~150 | 0.96(0.81-1.15) | 0.93(0.78-1.11) | 0.94(0.79-1.13) |
| 150~400 | 1.35(1.14-1.59) | 1.31(1.11-1.56) | 1.34(1.13-1.59) |
| ≥400 | 1.58(1.35-1.86) | 1.50(1.27-1.76) | 1.49(1.26-1.76) |
| *P_trend_* | <.001 | <.001 | <.001 |

**Note:** NSSI, non-suicidal self-injury; OR, odds ratio; CI, confidence interval.

Model 1, adjusted for gender, grade, race, registered permanent residence, siblings, and parental educational attainment.

Model 2, adjusted for gender, grade, race, registered permanent residence, siblings, parental educational attainment, current smoking, current drinking, physical activity, and rational diet.

Model 3, adjusted for gender, grade, race, registered permanent residence, siblings, parental educational attainment, current smoking, current drinking, physical activity, rational diet, family disasters, hospitalization experience, failed exams, and failed relationships.

**Table S3.** Associations between objectively measured smartphone use and NSSI by multilevel model with classes as random effects.

| Objectively measured smartphone use | *OR(95%CI)* | | |
| --- | --- | --- | --- |
|  | Model 1 | Model 2 | Model 3 |
| Smartphone screen time (hours/week) |  |  |  |
| 0~21 | 1[Reference] | 1[Reference] | 1[Reference] |
| 21~42 | 0.82 (0.52,1.30) | 0.80 (0.51,1.28) | 0.83 (0.52,1.32) |
| 42~63 | 1.06 (0.74,1.51) | 1.03 (0.73,1.47) | 1.03 (0.73,1.47) |
| ≥63 | 1.63 (1.19,2.23) | 1.55 (1.14,2.12) | 1.53 (1.12,2.09) |
| *P_trend_* | <.001 | <.001 | <.001 |
| Smartphone screen time, per 21 hours/week | 1.21 (1.12,1.30) | 1.19(1.10-1.29) | 1.18(1.09-1.28) |
| Number of smartphone unlocks (times/week) |  |  |  |
| 0~50 | 1[Reference] | 1[Reference] | 1[Reference] |
| 50~150 | 1.28 (0.81,2.02) | 1.18 (0.75,1.87) | 1.06 (0.67,1.67) |
| 150~400 | 1.69 (1.10,2.62) | 1.62 (1.05,2.48) | 1.46 (0.95,2.25) |
| ≥400 | 2.07 (1.35,3.18) | 1.93 (1.26,2.95) | 1.70 (1.11,2.61) |
| *P_trend_* | <.001 | <.001 | <.001 |
| Number of smartphone unlocks, per 50 times/week | 1.03(1.01-1.05) | 1.03(1.01-1.04) | 1.02(1.01-1.04) |

**Note:** NSSI, non-suicidal self-injury; OR, odds ratio; CI, confidence interval.

Model 1, adjusted for gender, grade, race, registered permanent residence, siblings, and parental educational attainment.

Model 2, adjusted for gender, grade, race, registered permanent residence, siblings, parental educational attainment, current smoking, current drinking, physical activity, and rational diet.

Model 3, adjusted for gender, grade, race, registered permanent residence, siblings, parental educational attainment, current smoking, current drinking, physical activity, rational diet, family disasters, hospitalization experience, failed exams, and failed relationships.

**Table S4.** Association between smartphone screen time and number of smartphone unlocks by Chi-square test.

| Smartphone screen time (hours/week) | Number of smartphone unlocks (times/week) | | | | *χ*² | *P* |
| --- | --- | --- | --- | --- | --- | --- |
|  | 0~50 | 50~150 | 150~400 | ≥400 |  |  |
| 0~21 | 1576(42.5) | 1307(35.3) | 454(12.2) | 370(10.0) | 3209.2 | <.001 |
| 21~42 | 628(22.3) | 1027(36.4) | 636(22.6) | 527(18.7) |  |  |
| 42~63 | 528(12.2) | 1190(27.5) | 1148(26.6) | 1456(33.7) |  |  |
| ≥63 | 458(7.9) | 1412(24.3) | 1941(33.3) | 2010(34.5) |  |  |

**Table S5.** Spearman correlation between smartphone screen time and number of smartphone unlocks.

|  | Smartphone screen time | Number of smartphone unlocks |
| --- | --- | --- |
| Smartphone screen time | 1 | .347^a^ |
| Number of smartphone unlocks | .347^a^ | 1 |

**Note:** ^a^ Results is statistically significant at *P*<.001
